# The sonographic characteristics of unicentric castleman disease - a single-center retrospective study

**DOI:** 10.1186/s40644-025-00937-2

**Published:** 2025-09-29

**Authors:** Zihan Liu, Zihan Niu, Yuhan Gao, Mengsu Xiao, Ying Wang, Qingli Zhu, Lu Zhang

**Affiliations:** 1 Department of Ultrasound, Peking Union Medical College Hospital, Chinese Academy of Medical Sciences and Peking Union Medical College, No. 1 Shuaifuyuan, Dongdan, Beijing, 100730 China; 2 Department of Hematology, Peking Union Medical College Hospital, Chinese Academy of Medical Sciences and Peking Union Medical College, No. 1 Shuaifuyuan, Dongdan, Beijing, 100730 China

**Keywords:** Unicentric castleman disease, Ultrasonography, Lymph node, Diagnostic imaging, Hyaline vascular subtype, Echo pattern

## Abstract

**Background:**

Unicentric Castleman disease (UCD) is a rare group of non-neoplastic lymphoproliferative disorders. This study aims to summarize the specific ultrasonic manifestations of UCD.

**Methods:**

This retrospective study included patients who underwent preoperative ultrasound for enlarged lymph nodes and were later diagnosed with UCD between January 2016 and March 2024. Ultrasound features, including lymph node size, cortical characteristics, corticomedullary interface, hyperechoic regions, and Doppler flow signals, were recorded. Pathological types were classified as hyaline vascular (HV), plasma cell (PC), or mixed. The ultrasonic features of each UCD subtype were systematically analyzed.

**Results:**

A total of 41 patients were enrolled in the study, comprising 29 with HV-type, 4 with PC-type, and 8 with a mixed type. All patients presented with enlarged lymph nodes (LNs) characterized by a solitary mass, well-defined margins, and increased cortical thickness. Among these, 95.12% (39/41) exhibited an indistinct corticomedullary interface. Additionally, 41.46% (17/41) showed eccentric or asymmetrical cortical thickening, while 58.54% (24/41) demonstrated complete effacement of the fatty hilum. Approximately 24.39% (10/41) of cases exhibited macrocalcification, and 56.10% (23/41) displayed short linear hyperechoic foci within the lymph nodes. Furthermore, patients with HV-type and mixed-type conditions exhibited more abundant blood flow signals compared to those with PC-type (75.86% vs. 25% vs. 87.50%, *P* = 0.018).

**Conclusions:**

Ultrasound characteristics of UCD generally comprise sizable, solitary masses with clearly delineated borders, a thickened cortex, and disappearance of the fatty hilum. Principal imaging indicators encompass microcalcifications and short linear hyper-echoes. Ultrasound represents an effective and non-invasive modality for the early identification and diagnosis of UCD.

**Trial registration:**

Retrospectively registered.

## Introduction

Castleman disease (CD) is a heterogeneous group of non-neoplastic lymphoproliferative disorders [1], first described by Castleman and Towne in 1954 in a patient presenting with a mediastinal mass [2]. Based on clinical manifestations and disease progression, CD is classified into unicentric CD (UCD) and multicentric CD (MCD). UCD accounts for approximately 75% of the newly emerging CD. It is characterized by a relatively benign clinical course, which is typical of a localized and indolent disease involving a single enlarged lymph node (LN) or multiple enlarged LNs within a single area [3, 4]. Surgical resection is the gold standard of treatment for UCD, providing a definitive diagnosis and surgical cure of the disease [4, 5]. However, UCD is relatively uncommon and does not demonstrate any specific clinical or laboratory findings [6, 7]; therefore, its preoperative diagnosis warrants a radiological evaluation. In particular, image-guided surgery is crucial for diagnosing and treating UCD.

Computed tomography (CT) of the neck, chest, abdomen, and pelvis is recommended as the initial imaging modality to assess lymph node involvement and differentiate unicentric (UCD) from multicentric (MCD) types during the diagnosis of Castleman disease (CD) [4]. Ultrasound, a convenient and versatile imaging examination method that does not involve radiation, is suitable for the rapid assessment of easily accessible areas, such as the neck, axilla, and groin. With the rapid development and widespread use, an increasing number of CDs are incidentally detected by the US during physical examinations. Especially for patients with symptoms of palpable masses, ultrasound is the preferred method [8]. However, there is limited data available for systematic analysis of CD ultrasound imaging characteristics. Most studies were case reports [9–11], and the largest number of UCD cases was 15 [12]. At present, the ultrasound findings of UCD are often described together with MCD, and there is no conclusion regarding the different pathological types, leading to a poor understanding of this disease.

We therefore conducted a retrospective analysis of patients with UCD transferred to our hospital, reviewing their US images based on a relatively large sample size. We aim to summarize the characteristic ultrasonic features of UCD, thereby enhancing diagnostic accuracy for clinicians.

## Materials and methods

### Study design and patients

This study was approved by the Institutional Review Board of Peking Union Medical College Hospital (Approval No: S-K1539), and the requirement for written informed consent was waived. Patients from Peking Union Medical College Hospital between January 2016 and March 2024 were included. The inclusion criteria were as follows: (1) Patients diagnosed with unicentric Castleman disease (UCD) based on complete lymph node resection biopsy and clinical features consistent with solitary enlarged lymph nodes; (2) Patients who had undergone preoperative ultrasound examination.

### Ultrasonic imaging

All US examinations were performed using Resona 7 or 8 devices (L 5–14, Mindray Medical, Shenzhen, China), Acuson S2000 (L 8–14, Siemens Healthcare, Erlangen, Germany), and the iU22 machine (L 5–12, Philips Healthcare, Amsterdam, Netherlands). Both 2D and color Doppler images were reviewed. Based on previous reports, the following US features were used to evaluate LNs. For multiple enlarged lymph nodes (LNs) within a single station, ultrasound (US) images of the biopsied LNs were assessed.

**Fig. 1:**
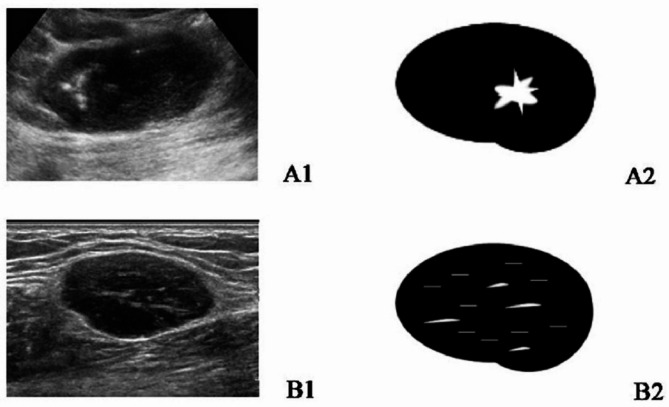
Two sonographic presenting patterns of unicentric Castleman disease. The grayscale US images (left) and the schematic illustration images (right) are shown in the figures **A **(**A1-A2**) Pattern I, the hypoechoic enlarged LN had a thickened cortex or hilum effacement. The macro strong echo with acoustic shadow was shown inside the node. **B** (**B1-B2**) Pattern II, the hypoechoic enlarged LN had a thickened cortex or hilum effacement. The short linear strong echo was visible inside the LN US, ultrasound; LN, lymph node

In grayscale ultrasound, lymph node size (long and short diameters), LN shape (regular, lobulated), margin (well-defined, obscure), cortex characteristics (eccentrical or asymmetrical thickened cortex and complete effacement of the fatty hilum), corticomedullary interface (clear, unclear), matting (yes, no), macrocalcification (yes, no), and short linear sign (yes, no) were recorded [13, 14]. Macrocalcification is defined as coarse hyper-echoic areas with an acoustic shadow. The short linear sign is a short, linear, strong echo within the LN (Fig. 1).

For Color Doppler imaging, the gain was standardized by initially increasing it until background noise became visible, followed by a slight reduction to eliminate noise and prevent overestimation of vascularity [13, 15, 16]. The color flow distribution (absent, hilar or non-hilar) [13] and the amount of blood flow signals (no color, minimal or abundant) were recorded. The color Doppler flow characteristics of the lesion were observed and semi-quantitatively classified into 3 types: no color, absence of blood flow signals; minimal blood flow, with minimal scattered color signals,1 to 2 punctate or thin linear vessels visible, the length of linear blood flow does not exceed half of the lesion’s diameter; abundant blood flow, the entire length of the vessel/vessels can be observed, with more than 4 punctate vessels or more than one longer vessel penetrating the lesion, its length can approach or exceed half of the mass’s radius [17].

### Pathological diagnosis of SLNs

Combined with clinical and imaging examinations, surgical resection or core biopsy was performed on patients with suspected lymphadenopathy (LN). Pathological findings helped classify the lesions into three subtypes of Castleman disease (CD): hyaline vascular subtype (HV), plasma cell subtype (PC), and mixed subtype. The HV subtype was identified by atrophic follicles with hyaline vessels and concentric rings of lymphocytes, along with a predominance of lymphocytes in the interfollicular areas. The PC subtype was characterized by the absence of follicular hyaline vessels in LN structures and notable aggregation of plasma cells in the interfollicular zone. Lesions showing both HV and PC features were classified as the mixed subtype [18].

### Statistical analyses

The continuous variable was shown as the mean ± standard deviation and analyzed by an independent two-sample t-test. Categorical variables were expressed as cases and percentages and analyzed by the chi-square test or Fisher’s exact test. *P* < 0.05 was considered significant. The software Image J was used to calculate the long and short diameters of LN and the thickness of the cortex. Statistical analysis was performed using SPSS 26 software.

## Results

### Patient characteristics of the cohorts

A total of 41 patients were ultimately included in this study, comprising 25 females (60.98%) and 16 males (39.02%). The mean age of the patients was 34.34 years, with a standard deviation of 13.57 years. Regarding clinical manifestations, 36.59% (15/41) of UCD patients were asymptomatic and incidentally detected during imaging examinations, while 46.34% (19/41) had palpable masses. 56.10% (23/41) of cases involve superficial lymph nodes, including inguinal (*n* = 2), axillary (*n* = 5), and cervical (*n* = 16) areas. Additionally, 73.17% (30/41) of patients exhibited single LN enlargement at a single station, whereas 26.83% (11/41) of patients had multiple LN enlargements at a single station. Detailed clinical baseline characteristics of the enrolled patients are summarized in Table 1.

**Table 1:** The clinical baseline of UCD patients

Clinical characteristics	UCD
	(*n* = 41)
Age	34.34 ± 13.57
Gender	
Female	25(60.98)
Male	16(39.02)
Histopathology	
Hyaline vascular type (HV)	29 (70.73)
Plasma cell type (PC)	4(9.76)
Mixed type	8(19.51)
Clinical manifestations	
By palpation at physical examination	19(46.34)
Compression symptoms	7(17.07)
Incidentally discovered by imaging	15(36.59)
Distribution	
superficial	22(53.66)
deep	19(46.34)
Lymph node distribution	
Single enlarged LN	30(73.17)
Multiple enlarged LNs in one area	11(26.83)
Lymph Node Location	
Retroperitoneal	11(26.83)
Abdominal pelvic cavity	7(17.07)
Inguinal	2(4.88)
Axilla	5(12.20)
Cervical	16(39.02)

### US findings for UCD and image analysis

**Fig. 2:**
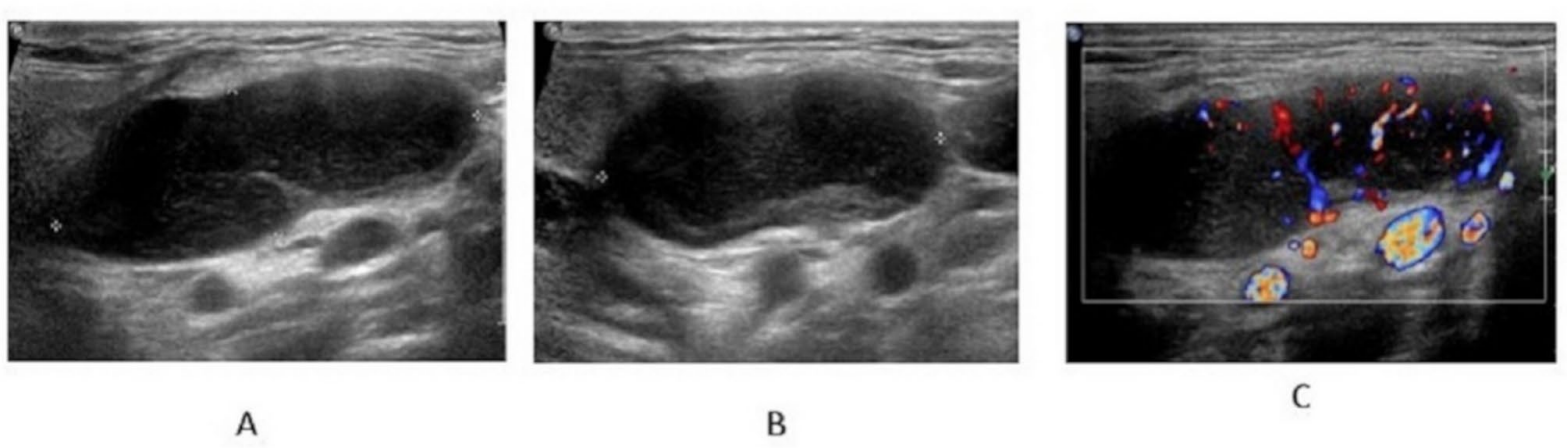
US images from a 36-year-old man with a palpable mass in the left cervical region. The pathological diagnosis of the enlarged LN was the hyaline vascular type (**A-B**) The enlarged cervical lymph node grayscale US. The size of the lymph nodes was 4.7 × 3.4 × 1.7 cm. The LN showed a thickened cortex. Short linear hyperechoic areas are visible within the LN. (**C**) The color Doppler image. The LN showed abundant hilar color flow US, ultrasound; LN, lymph node; cm, centimeter

The ultrasound findings for all patients are summarized in Table 2. The median long diameter of the enlarged lymph nodes was 4.37 cm (range, 1.9–8.7 cm), while the median short diameter was 2.46 cm (range, 0.6–6.6 cm). The average L/S (long/short) diameter ratio was 1.99. All patients exhibited enlarged lymph nodes characterized by solitary, well-defined margins, regular shape, and increased cortical thickness. Notably, 95.12% (39/41) of the lymph nodes demonstrated an indistinct cortico-medullary interface. Eccentric or asymmetrical thickening of the cortex was observed in 41.46% (17/41) of the lymph nodes (Fig. 2), and 58.54% (24/41) of the lymph nodes showed complete effacement of the fatty hilum. Specifically, 24.39% (10/41) of patients exhibited macrocalcification (Pattern I), and 56.10% (23/41) displayed short linear hyperechoic foci (Pattern II). A total of 73.17% (30/41) of patients had the characteristic distribution patterns.

**Table 2:** The ultrasound characteristics of all patients with UCD

Ultrasound characteristics	UCD
	(*n* = 41)
Long axis (length, L) (cm)	4.37 ± 1.52
Short axis (depth, S) (cm)	2.46 ± 1.20
Long/short-axis ratio (L/S ratio)	1.99 ± 0.77
L/S ratio < 2.00	22
L/S ratio ≥ 2.00	13
L/S ratio ≥ 3.00	6
Nodal shape	
Oval	33(80.49)
Lobulated	8(19.51)
Cortex thickness(cm)	2.12 ± 1.26
Cortex characteristic	
Eccentric or asymmetrical thickened cortex	17(41.46)
Complete effacement of fatty hilum	24(58.54)
Corticomedullar interface	
Clear	2(4.88)
Unclear	39(95.12)
Macrocalcification	
0	31(75.61)
1	10 (24.39)
Short linear hyperechoic foci​	
0	18(43.90)
1	23(56.10)
Either macrocalcification or short linear hyperechoic foci	30(73.17%)
Both macrocalcification and short linear hyperechoic foci​	3(7.32%)
Blood distribution	
None	4(9.76)
Hilar color flow	3(7.32)
Non-Hilar color flow	34(82.92)
Blood volume	
No color	4(9.76)
Minimal	7(17.07)
Abundant	30(73.17)

**Fig. 3:**
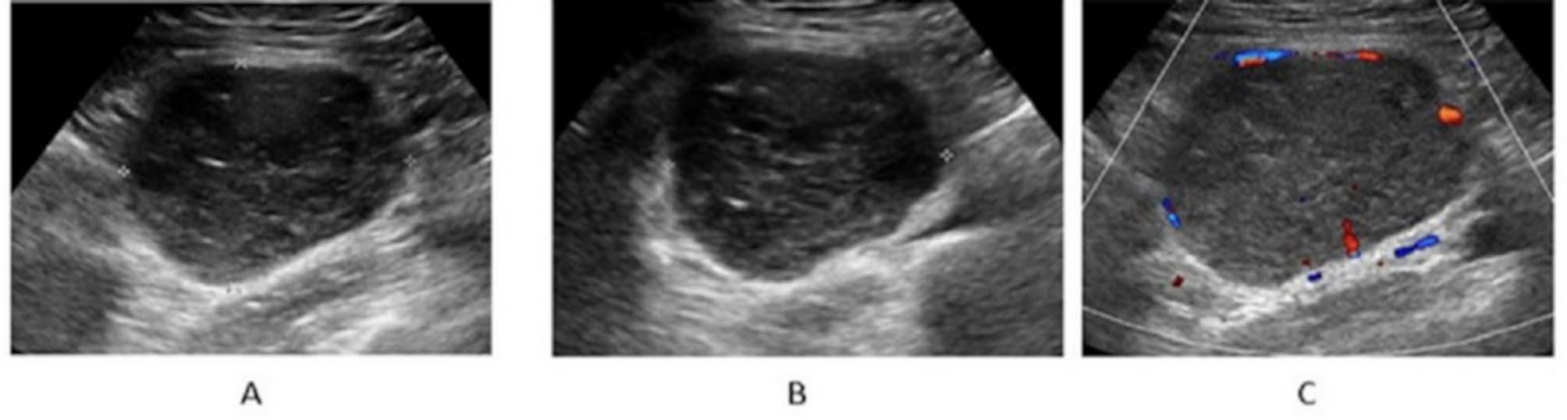
The US findings of an enlarged LN in a 50-year-old woman with a palpable mass in the right lower quadrant. The pathological diagnosis of the enlarged LN was the mixed type (**A- B**) The enlarged lymph node grayscale US. The retroperitoneal lymph node presented 7.8 × 7.2 × 6.6 cm in size. The LN showed complete effacement of the fatty hilum and a short linear hyper-echo within the LN. (**C**) The Color Doppler image. The LN showed moderate non-hilar color flow US, ultrasound; LN, lymph node; cm, centimeter

**Fig. 4:**
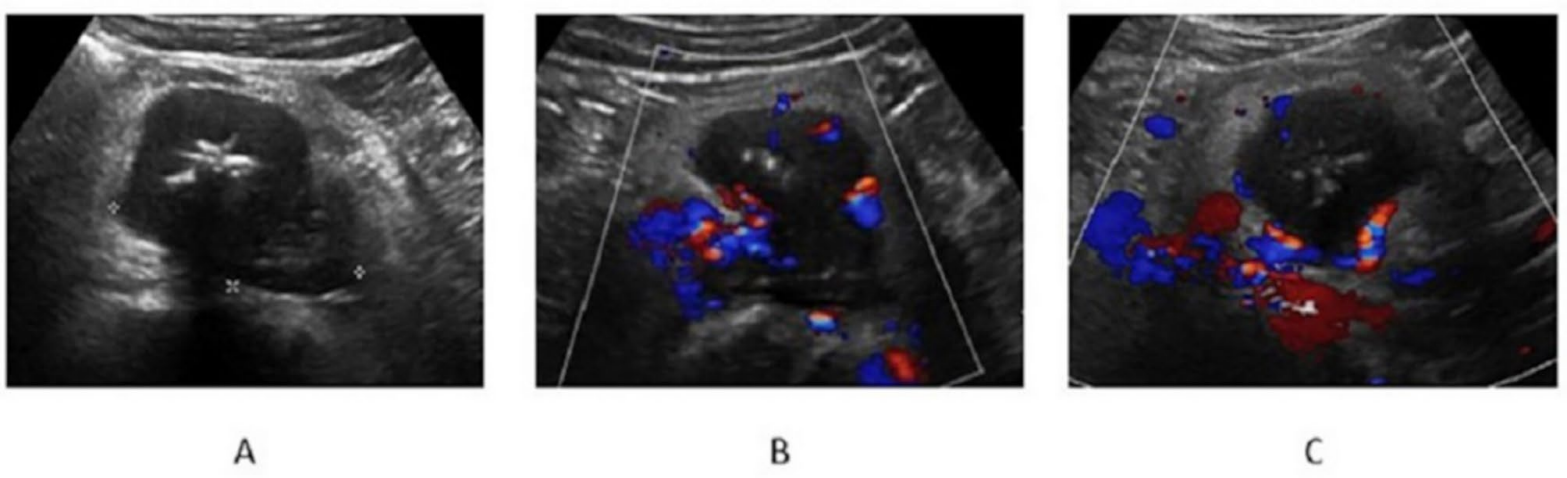
US images from a 36-year-old man with a palpable mass in the right lower quadrant. The pathological diagnosis of the enlarged LN was the mixed type (**A- B**) The enlarged lymph node grayscale US. The retroperitoneal lymph node presented 6.1 × 5.1 × 4.4 cm in size. A macro strong echo with the posterior shadow is visible in the enlarged LN. (**C**) The color Doppler image. The LN showed abundant hilar color flow US, ultrasound; LN, lymph node; cm, centimeter

Color Doppler imaging revealed increased blood flow in 73.81% (30/41) of the lymph nodes, with a non-hilar color pattern observed in 82.92% (34/41) of cases. The US images of 2 representative cases are shown in Figs. 3 and 4.

### US findings for HV, PV, and HVPC and image analysis

According to the pathological results, 70.73% (29/41) of the patients were classified as hyaline vascular type (HV), 9.76% (4/41) as plasma cell type (PC), and 19.51% (8/41) as mixed type. The US findings of different pathological types were shown in Table 3 (note that percentages may not sum to 100% due to rounding). 75.86% (22/29) of HV patients and 87.50% (7/8) of mixed-type patients exhibited enlarged lymph nodes with abundant blood flow, while only 25.00% (1/4) of PC patients showed rich blood flow, indicating a statistically significant difference (*P* = 0.018).

When comparing HV and non-HV types, statistically significant differences were observed in cortical features (*p* = 0.035). In the HV group, 68.97% (20/29) of patients demonstrated complete effacement of the fatty hilum, and 33.33% (4/12) showed thickened cortex. When comparing HV and PC types, significant statistical differences were noted in both blood volume and cortical characteristics. For the HV group, 75.86% (22/29) of patients exhibited abundant lymph node blood flow, whereas 25.00% (1/29) of patients in the PC group showed rich blood flow signals (*P* = 0.007).

**Table 3:** US findings for HV, PV, and HVPC

Ultrasound characteristics	Pathology			P* *value*	*P*† *value*	*P*‡ *value*
	HV (*n* = 29)	PC (*n* = 4)	Mixed (*n* = 8)			
Cortex characteristic				0.077	0.451	0.035**
Eccentric or asymmetrical thickened cortex	9(31.03)	2(50.00)	6(75.00)			
Complete effacement of fatty hilum	20(68.97)	2(50.00)	2(25.00)			
Macrocalcification				0.226	0.133	0.098
No	24(82.76)	2(50.00)	5(62.50)			
Yes	5(17.24)	2(50.00)	3(37.50)			
Short linear hyperechoic foci​				0.88	0.744	0.613
No	12(41.38)	2(50.00)	4(50.00)			
Yes	17(58.62)	2(50.00)	4(50.00)			
Blood distribution				0.303	0.306	0.971
None	3(10.34)	1(25.00)	0(0.00)			
Hilar color flow	2(6.90)	1(25.00)	0(0.00)			
Non-Hilar color flow	24(82.76)	2(50.00)	8(100.00)			
Blood volume				0.018**	0.007**	0.112
No color	4(13.79)	0(0.00)	0(0.00)			
Minimal	3(10.34)	3(75.00)	1(12.50)			
Abundant	22(75.86)	1(25.00)	7(87.50)			

* *P* values compare all three groups

† *P* values compare hyaline vascular type (HV) and plasma cell type (PC)

‡*P* values compare hyaline vascular type (HV) and non-hyaline vascular type

** *P* <0.05

## Discussion

This paper evaluated the ultrasound features of UCD based on a larger dataset, contributing to a deeper understanding of the disease in clinical practice. Our results demonstrated that the UCD usually exhibited enlarged lymph nodes with complete effacement of the fatty hilum and unclear corticomedullary interface. 73.17% of patients showed a strong echo, including either macrocalcification or short linear hyperechoic foci within the LNs, which were notably characteristic findings for UCD. In addition, compared with the PCV, the HV groups exhibited more abundant blood flow signals for lymph nodes.

In our study, the majority of UCDs were detected incidentally by physical examination or medical visits for palpable masses. UCD classically presents as hypoechoic masses with increased volume and cortical thickening, which is associated with pronounced follicular hyperplasia in the cortical region and atrophic germinal centers [18]. Additionally, the reactive proliferation of B cells and T cells within the cortex may further contribute to cortical thickening [19]. The macrocalcifications and short linear strong echoes in the LNs were characteristic findings in this study. The macrocalcifications on US appear as echogenic shadowing foci with discrete, coarse, arborizing configurations [20–22]. The presence of calcification may indicate a diagnosis of UCD [23–25]. Calcification may be due to the calcium deposition occurring based on the specific pathology changes, including thickening of the proliferative capillary wall, accompanied by hyaline degeneration, fibrinolysis, and degeneration of other connective tissues.

This study describes the observation of central short linear echogenic foci within unicentric Castleman disease (UCD) lesions. This ultrasonographic feature, observed in 56.10% of cases in our cohort, suggests considerable diagnostic value for UCD. Histopathologically, the echogenic foci are hypothesized to correspond to the proliferative fibrovascular stroma and hyalinized vessels that characteristically surround the hyperplastic lymphoid follicles in UCD, particularly the hyaline-vascular subtype [26, 27]. The underlying tissue alterations of fibrosis and architectural distortion can also manifest as similar sonographic patterns in other conditions. For instance, they may be observed in treated lymphoma (as a result of post-therapeutic fibrotic scarring) [28], granulomatous diseases such as sarcoidosis (due to coalescing granulomas and connective tissue proliferation) [29], and occasionally in metastatic lymph nodes (with desmoplastic stromal reactions) [27, 30]. Therefore, the short linear echogenic foci must be interpreted in conjunction with other sonographic features. A solitary, well-defined, markedly hypervascular mass with cortical thickening and echogenic foci is highly suggestive of UCD [26].

For different CD histological types, HV-type patients generally exhibit combined blood flow patterns and abundant flow signals. According to a previous study, after the administration of a contrast agent, the enhancement degree of the PV type is usually lower than that of the HV type [20]. The blood flow perfusion of UCD was consistent with our results, which is due to the proliferation and expansion of the capillary network around the lymphoid follicles in the HV subtype, while in PV subtype lesions, the diffuse plasma cell infiltration and fibrotic tissue compress or occupy normal vascular spaces, increasing vascular resistance and reducing blood flow. The mixed subtype is characterized by a pattern that may depend on whether hyaline vascular changes or plasma cell infiltration is more predominant in the pathological process [18].

The diagnosis of CD is often suspected initially owing to radiographic findings. In this study, we propose some imaging signs to provide auxiliary guidance for physicians during clinical procedures. In particular, the two characteristic patterns may be helpful for the diagnosis of CD. However, accurate preoperative diagnosis of CD is difficult based on radiologic features alone. The US findings can be manifestations of several diseases, including lymphoma, metastatic LN, and other diseases. Future research will therefore require considerably larger cohorts to investigate any difference.

Our study has some limitations. First, it is a single-center retrospective study; due to the low incidence of UCD, the number of cases included in this study was relatively limited. As our hospital is a comprehensive tertiary hospital for the rarity of CD, our study includes the largest UCD cohort to date. Second, for some deep-seated LNs, the richness of blood flow might be underestimated due to the application of a relatively low-frequency probe. Future prospective studies need to be conducted with a standardized diagnostic workup.

## Conclusions

UCD is typically manifested as a solitary enlarged lymph node with a thick cortex, absence of medullary structure, and an unclear cortico-medullary demarcation. These lesions showed macrocalcification or short linear hyperechoic patterns. They may indicate a diagnosis of UCD. Additionally, compared with PV, the HV group presented more often with abundant color flow signals on color Doppler flow imaging. Ultrasound is expected to play a significant role in the evaluation of UCD and in guiding appropriate regions for biopsies.

## Data Availability

The datasets generated or analyzed during the study are available from the corresponding author on reasonable request.

## Abbreviations

CD: Castleman disease
UCD: Unicentric Castleman disease
MCD: Multicentric Castleman disease
HVV: Hyaline vascular variant
PCV: Plasma cell variant
HVPC: Hyaline-vascular plasma cell variant
US: Ultrasound
